# Systematic review of patient safety interventions in dentistry

**DOI:** 10.1186/s12903-015-0136-1

**Published:** 2015-11-28

**Authors:** Edmund Bailey, Martin Tickle, Stephen Campbell, Lucy O’Malley

**Affiliations:** 1 NIHR Greater Manchester Primary Care Patient Safety Translational Research Centre, Institute of Population Health - Centre for Primary Care, University of Manchester, 7th Floor: Williamson Building, Manchester, M13 9PL UK; 2 School of Dentistry, University of Manchester, J R Moore Building, Oxford Road, Manchester, M13 9PL UK

**Keywords:** Patient harm, Primary health care, Oral surgery, Health services research, Standard of care, Epidemiology

## Abstract

**Background:**

The concept of patient safety in dentistry is in its infancy, with little knowledge about the effectiveness of tools or interventions developed to improve patient safety or to minimise the occurrence of adverse events.

**Methods:**

The aim of this qualitative systematic review was to search the academic and grey literature to identify and assess tools or interventions used in dental care settings to maintain or improve patient safety. All study designs were included from all dental care settings. Outcome measures were: patient safety, harm prevention, risk minimization, patient satisfaction and patient acceptability, professional acceptability, efficacy, cost-effectiveness and efficiency. Quality assessments were performed on the included studies based on CASP tools. Further analysis was undertaken to discover whether any of the tools had been trialled or verified by the authors, or by subsequent authors.

**Results:**

Following abstract screening, and initial qualitative synthesis, nine studies were found to meet the inclusion criteria with 31 being excluded following initial analysis. Tools identified included: checklists (4 studies), reporting systems (3), the use of electronic notes (1) and trigger tools (1). Grey literature searching did not identify any further appropriate studies. In terms of study design, there were observational studies including audit cycles (5 studies), epidemiological studies (3) and prospective cluster randomised clinical trials (1). The quality of the studies varied and none of their outcomes were verified by other researchers. The tools identified have the potential to be used for measuring and improving patient safety in dentistry, with two surgical safety checklists demonstrating a reduction in erroneous dental extractions to nil following their introduction. Reporting systems provide epidemiological data, however, it is not known whether they lead to any improvement in patient safety. The one study on trigger tools demonstrates a 50 % positive predictive value for safety incidents. It is not clear as to what impact the introduction of electronic guidelines has on patient safety outcomes.

**Conclusions:**

This systematic review finds that the only interventions in dentistry that reduce or minimise adverse events are surgical safety checklists. We believe this to be the first systematic review in this field; it demonstrates the need for further research into patient safety in dentistry across several domains: epidemiological, conceptual understanding and patient and practitioner involvement.

**Electronic supplementary material:**

The online version of this article (doi:10.1186/s12903-015-0136-1) contains supplementary material, which is available to authorized users.

## Background

The US Institute of Medicine’s report ‘To Err is Human’ [1] shocked the medical profession by revealing that more people died in the USA as a result of medical error than from road traffic accidents. Following this report, there has been a growing emphasis placed on patient safety in all healthcare services including primary healthcare settings; it is known that the majority of patient contact with healthcare professionals occurs in primary care settings [2]. Recent high profile reports from the UK have demonstrated that some hospitals have failed to ensure that patient safety is maintained, raising awareness of the issue of patient safety amongst the public, politicians, clinicians and managers [3, 4].

Several definitions of ‘patient safety’ exist; [1, 4–9] these include the World Health Organisation’s (WHO) 2011 statement that patient safety relates to ‘The reduction of the risk of unnecessary harm associated with health care to an acceptable minimum’ [8]. The WHO defines unsafe patient care as a process or act of omission or commission that resulted in hazardous healthcare conditions and/or unintended harm to the patient [10]. In patient safety research, a framework of definitions, concepts and methods applicable to both primary and secondary care exist, and were compiled into a taxonomy in 2009 [11]. This document, also published by the WHO, describes in detail some of the most frequently used terminology relating to patent safety. It includes a discussion about ‘adverse events’. These are defined as unintended events occurring during the care process that resulted in, could have resulted in or may in the future result in actual harm to the patient. A preventable adverse event is described as an adverse event that would not have occurred if the patient had received ordinary standards of care appropriate for the time. Unpreventable adverse events describe events that result from a complication that cannot be prevented given the current state of knowledge. Only preventable adverse events can be used to reflect sub-optimal patient care in relation to patient safety. ‘Near misses’ are also discussed; these describe an event or situation in which medical error could have resulted in accident, injury, or illness, but did not; either by chance or through timely intervention [11].

A large volume of epidemiological data has been collected on medical error in hospital settings [12, 13]; however, less is known about patient safety in relation to primary care services. It is estimated that between 5 and 80 patient safety incidents occur per 100,000 consultations in primary medical care settings, with approximately 11 % of prescriptions containing errors [14]. A recent systematic review of interventions (or tools) used in primary medical care, to maintain or improve patient safety, found that the majority of these were designed to prevent adverse drug reactions. The authors noted a lack of patient participation in the included studies [15]. Further research is necessary to provide clarity in the primary medical care field [16]. Similarly, patient safety in primary care dentistry is an area where the evidence base is lacking at present [17, 18].

Toolkits have previously been used to improve patient safety in hospital settings [19], including the use of safety checklists [13]. However, we have a poor understanding of how patient safety in dentistry differs from other fields of healthcare and there have been no systematic reviews of patient safety in dentistry to provide a comprehensive understanding of the current knowledge base, or to identify existing tools designed to improve patient safety in dentistry [20].

Epidemiological data from studies relating to dentistry are uncommon. One recent review from the Netherlands [21] used systematic retrospective analysis to review the electronic records for any patients where a potential adverse event was identified. The researchers analysed 1000 records that were made up of 50 patients from the 20 practices that participated in the study; this amounted to 13,615 patient contacts over the 5-year period of analysis. The authors found that 18 adverse events had occurred; three were judged as being potential adverse events (or near misses), with the remaining 15 considered preventable. These adverse events included: one erroneous extraction, four cases of retained roots following tooth extractions, eight cases relating to endodontic therapy (including fractured instruments, perforations and leakage of sodium hypochlorite into the apical tissues) and two cases of crowns being swallowed by patients. The three ‘near misses’ were all in relation to radiographs not being taken prior to third molar extractions. A 2012 review [22] examined the United Kingdom NHS National Patient Safety Agency (NPSA) database and identified incident reports that related to dentistry and/or dental interventions. They found that during 2009, 36 cases of wrong tooth extraction were reported; 16 of these occurred when the patient was under a general anaesthetic. However, these figures relate entirely to secondary care (hospital) settings.

We believe this systematic review to be both important and timely in light of heightened political, clinical and patient interest in patient safety, and in order to add to the evidence base for patient safety interventions specific to dentistry.

## Methods

### Aims and objectives

The aim of this systematic review was to search the academic and grey literature, to identify and assess tools used in dental care to improve patient safety. The objectives of the review were to:

1. Find any systematic reviews on patient safety for dentistry,
2. identify any tools used within dentistry to assess the risk of harm to patients,
3. ascertain what tools have been used to reduce the risk of harm to patients receiving dental care,
4. discover whether any of the tools identified had been validated; either by the authors themselves, or by other researchers. We were looking specifically at whether these tools actually led to any improvement in patient safety outcomes.

We limited our search results to studies written in English. This study is a systematic review of previously published data and as such did not require ethical approval, as we used no human subjects in the preparation of this manuscript. This systematic review adheres to the PRISMA checklist for reporting systematic reviews (see Additional file 1).

### Inclusion Criteria for this Systematic Review

#### Types of studies

A comprehensive search was undertaken to identify both published and unpublished reports of studies that use, or describe the development of, patient safety interventions relating to dental care. Studies of all designs including descriptive, observational, and experimental methodologies were included in the review. We also included guidelines and systematic reviews in the search along with grey literature. Relevant organisations such as dental associations, protection societies, insurance companies, policy makers, and regulatory bodies were contacted as well as individuals known to have an interest in patient safety in dentistry to identify documents for inclusion. The review was confined to studies published after 1989 as we felt that culture and the nature of patient safety research was different prior to this. The Medical Subject Heading (MeSH) terms for the literature search were informed by a systematic review of patient safety in primary medical care [23] and adapted for dentistry by an Information Specialist; see Additional file 2 for the terms used and databases searched.

#### Participants and settings

As the authors believed there was a scarcity of previous research in this field, the review included all areas of dental practice including its specialities; covering both primary and secondary care settings. The review included care provided to all groups of patients. Please note that as this review was secondary research, there was no need to gain consent from any participants.

#### Interventions

Any pre-existing tools or interventions relating to the maintenance or improvement of patient safety outcomes in dentistry were included. All included studies were citation matched through Google Scholar to identify associated studies with the aim of establishing whether or not other authors had independently validated the tools or interventions. The studies were grouped according to the type of tool described or trialled.

#### Outcome measures

With regard to those studies testing tools or interventions, the outcome measures examined were patient safety, harm prevention, risk minimization, patient satisfaction and patient acceptability, professional acceptability, efficacy, cost-effectiveness and efficiency.

#### Data collection and analysis

All the titles and abstracts identified by the electronic search were downloaded to a reference management database. Duplicate entries were identified and removed. At least two review authors independently screened the titles and abstracts obtained from the initial electronic searches. Full text reports for the studies that fulfilled the inclusion criteria were identified. If sufficient information was unavailable in the study title or abstract to assess whether a study fulfilled the inclusion criteria, or if the abstract indicated that the study might be suitable for inclusion, the full published text was obtained and independently assessed in duplicate. Disagreement was resolved by discussion. The full texts were subjected to initial qualitative synthesis by at least one author; those found not to entirely fulfil the inclusion criteria at this stage were excluded with reasons for their exclusion provided. Studies that fulfilled the inclusion criteria at this stage were included in the full qualitative synthesis.

#### Quality Assessment

All of the included studies underwent a quality assessment based on the various CASP tools available for critical appraisal [24]. This assessment was performed by three of the authors and any disagreements were discussed in meetings. Any studies that were found to have methodological deficiencies were excluded at this stage.

## Results

### Included Studies

**Fig. 1:**
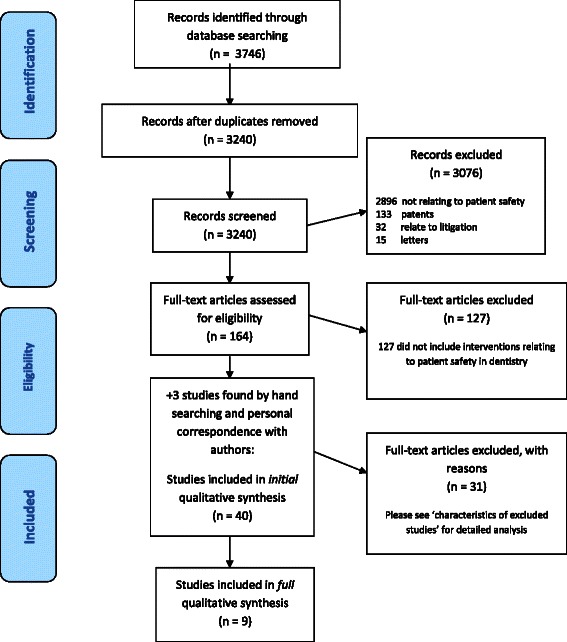
PRISMA Flow Diagram

The initial search identified 3240 published studies. These were then divided equally between four authors who screened the abstracts based on the inclusion criteria above. The 164 papers identified in the initial screening were discussed by the authors with the options of rejection, or possible inclusion into the review. At this point, 127 papers were rejected, with 37 for possible inclusion and further qualitative analysis. After finding the full texts for the 37 papers, one further study was found by searching through the citations of the included papers, and two very recently published studies were sourced via correspondence with the authors, making a total of 40 papers for initial qualitative synthesis (See Fig. 1).

The 40 included full text papers were then analysed and the following data extraction tool was applied:

- Type of study (RCT, observational etc.),
- Tool used? (yes/no: if no then reject),
- Primary/Secondary Care,
- What is the tool?
- What is the tool developed from? (for example the WHO checklist)
- Is there evidence of the tool being used?
- Has the tool been verified by others?
- Has the tool led to any improvements?

After further analysis and meetings with the author team, only nine of the studies were found to fit the inclusion criteria. The 31 excluded studies [17, 25–54], along with justifications for exclusion, are presented in Additional file 3. The nine included studies were [51, 55–62]. The characteristics of these studies are shown in Table 1. Please note that the original search was performed on 28.2.2014; however, six further articles were subsequently identified via the peer review process and these were also analysed and included in Fig. 1. The search for unpublished works (grey literature) did not yield any studies suitable for inclusion in this review.

**Table 1:** Tools used in included studies

Tool/intervention	Number of studies	Author & year of publication
Checklist	4	Lee 2007, Perea-Perez 2011, Saksena 2014, Beddis 2014
Reporting system	3	Lygre 2003, Scott 2004, van Noort 2004
Use of electronic notes	1	Fricton 2011
Trigger tools	1	Kalenderian 2013

### General Description of Included Studies

The nine included studies were found to have different outcome measures and therefore different measures of success. These findings are summarised in Table 2.

**Table 2:** Outcome measures

Studies that assess or detect the presence of adverse events	Perea-Perez 2011
	Lygre 2003
	Scott 2004
	Van Noort 2004
	Fricton 2011
	Kalenderian 2013
Studies with interventions that were used to prevent, minimise or reduce adverse events	Saksena 2014
	Lee 2007
	Beddis 2014

### Use of checklists

One recently published study [62] documented the introduction of a correct site surgery checklist to the dental division of Central Manchester University Hospitals for outpatients undergoing dental extractions under local anaesthesia, with or without sedation. The original checklist was introduced to the division in January 2009; compliance with this checklist was poor, as revealed by audit cycles. In 2012, a revised checklist was introduced based largely on the WHO checklist. Clinical staff were engaged in the process of developing the policy and training on the use of the checklist was provided to all members of staff and students in the division. Following the introduction of the 2012 checklist policy, audit revealed a 100 % compliance with the checklist. Importantly, no wrong tooth extractions have occurred in the 24 months since the checklist was introduced. Prior to this, during the period from 2009 to 2012, five cases of wrong tooth extraction had occurred in the division.

Lee and colleagues [57], in an educational piece, advocated the development of an educational programme to minimise the number of wrong tooth extractions occurring. The proposals were three pronged; an educational programme, a pre-operative checklist and an informative unambiguous referral form. It is important to note that this research was completed before the creation of the WHO surgical safety checklist [63]. The authors suggest that one way of reducing error is to simplify the processes involved. Their new referral form for practitioners is very straightforward, with a schematic diagram of the mouth for the practitioner to mark with an ‘x’ the tooth/teeth for removal. Following introduction of their guidelines for preventing wrong tooth/wrong site surgery, the authors observed a reduction in the number of wrong tooth extractions from 5 over a two-year period (before implementation of the policy) to nil over a one-year period post-implementation.

A team based in Madrid [59] reported a proposal for an 18 point surgical safety checklist for use in patients attending for ambulatory oral surgery procedures. The paper did not give details as to whether adoption of the checklist has led to any decrease in the number of wrong site procedures occurring in their department.

Another recently published study demonstrates the use of a safety checklist for patients attending a specialist temporomandibular joint (TMJ) disorder clinic in Manchester [51]. In this study, the authors describe a checklist that is used to identify patients with trismus, who are suffering from malignancy rather than TMJ related disorders, whilst attending a clinic for TMJ disorders. The checklist is used when assessing patients who present with trismus. The following diagnostic criteria will trigger a review with a senior clinician and other investigations as appropriate:

- Mouth opening of less than 15 mm,
- Progressively worsening trismus,
- Absence of a history of clicking,
- Pain of non-myofascial origin,
- Lymphadenopathy,
- Presence of suspect intra-oral soft tissue lesions.

The authors describe two cases where misdiagnoses were made prior to the correct diagnoses of malignancy. The checklist was introduced and one patient is subsequently described as having been identified as having a malignancy via the use of this checklist. The authors conclude that their checklist can be used to assist in the assessment of patients with trismus, in order to avoid misdiagnosis of more sinister underlying pathology.

The four papers described are observational studies; three are before and after studies and one is an educational piece; they do not make use of control groups. There are clear statements of the findings from each study and all of the papers include a discussion of the current policies and available literature. However, due to the lack of control groups, their findings must be interpreted with caution.

### Reporting Systems

We identified three papers that looked at the use of reporting systems in dentistry [58, 60, 61]. These papers have some overlap of authorship and are based around reporting systems for adverse reactions to dental materials.

Lygre and colleagues [58] reported on a Norwegian National reporting system established in 1993. The aim was to establish an adverse reaction registry and to serve as a clinical unit for patients affected by reactions to dental materials. It was a voluntary reporting system, with amalgam found to be the material most frequently causing adverse reactions (50 %). It is not known whether this study led to any improvements in patient safety as it is an epidemiological paper detailing the number of cases of adverse reaction, by type over a six year period. This paper is frequently cited, although no subsequent studies have validated their findings. The authors published a further paper two years later [64], which demonstrated a reduction in symptoms in the patients who had restorations replaced following adverse reactions.

The remaining two papers are from the UK. Scott and colleagues [60] sent reporting forms to dentists (27,000) and dental labs (2,700) encouraging responses. A postal survey of 1,000 dentists also took place as authors suspected under reporting. Amalgam was the most frequent cause of reaction for patients, with rubber products and resins affecting dental staff and technicians respectively. Again, this was an epidemiological paper with figures presented over a three year period of reporting. Van Noort and colleagues [61] discuss the systems in place in the UK, Norway & Sweden for reporting adverse reactions to dental materials. Again, it is an epidemiological paper with details of 1,268 adverse reaction reports from Norway, 848 from Sweden and 1,117 from the UK. Their findings echo both the Scott and the Lygre studies in that the most frequent cause of adverse reaction being metals (including amalgams) in patients, and rubber products in dental professionals. They also found that there are no standardised criteria as to what constitutes an adverse reaction to a dental material; and they also believed that under-reporting was an issue. The authors acknowledged that it takes a considerable amount of time for a pro-active reporting system to be established.

Through contact with the lead author (van Noort), it was established that the reporting system is no longer in use due to a limited funding period, which has since expired.

The three papers analysed here are epidemiological studies with similar outcomes assessed along with similar results. There has been sufficient analysis of the data found and there are clear statements of findings and discussion around contemporary literature and standards.

### Use of Electronic Notes

Fricton [55] trialled an intervention that alerted the practitioner to web-based guidelines regarding their patient’s medical conditions. In this three-arm, prospective, cluster randomised clinical trial, two approaches were used to engage with practitioners. In the first, a flashing alert was generated during the visit on the electronic notes system inviting the practitioner to look at the current guidelines relating to the patient’s medical conditions. In the second approach, patients who participated in the study were required to ask their dentist to review the care guidelines specific to their medical conditions; a control group was also used. Four medical conditions were included: xerostomia, diabetes, chronic obstructive pulmonary disease (COPD) and congestive heart failure. The authors found that the rate at which the practitioners accessed the guidelines increased during the first six months, but that by the end of the study period (18 months), the rate of use of guidelines had returned to the baseline levels. This may have been due to the practitioners not feeling the need to continue reviewing the guidelines, as they may have committed them to memory during the initial six month period, although this hypothesis was not tested.

The trial did address a clearly focused issue, which was the use of a clinical decision support tool integrated into electronic notes. Cluster randomisation was used to allocate the clinics into the three arms of the trial; this is an appropriate method for this type of trial and all providers were ‘blinded to’ the study protocol. The participating practices were compared and found to be similar in terms of the number of people working in them, the number of patients seen during the study period, and the number of patients with the medical conditions being studied. Other than the intervention, the three groups were treated the same. Several outcomes were measured with the key outcomes being the number of website hits per dental care provider, the percentage of dental care providers in each group accessing the online guidelines at the point of care and the proportion of providers who continued to access the guidelines throughout the study period. The authors found the statistically significant outcomes (*P* < 0.05) to be an increase in the use of guidelines for all patients even if they were not part of the study, and that provider activation was more effective than patient evaluation. These results do suggest a precise estimate that the intervention was successful.

The authors mention some significant limitations in the study; these were that 50 % of the hits recorded were during appointments with patients who were not included in the study (due to their funding source), and there may have been some crossover between study arms as a number of providers worked at more than one clinic included in the trial. It was also thought that there may have been crossover of patients between clinics. These issues mean that the study was prone to bias in terms of allocation and blinding of both patients and providers.

The effect of clinicians accessing these guidelines, or whether they have any impact on patient safety outcomes, remains unclear.

### Trigger Tools

A trigger is an easily detectable, focused item in a patient’s case notes that can help to lead to the identification of an adverse event. Triggers can inform examination of case notes to find out whether an adverse event actually occurred [65]. An example of a trigger is administration of naloxone, a drug that acts as an antagonist to opioids. The use of naloxone implies that there has been an overdose of opioids that would be considered an iatrogenic adverse event in the absence of drug abuse or self-inflicted overdose.

One paper [56] discusses the use of trigger tools in dentistry. This paper is from the USA and the authors compared the performance of an ‘Outpatient Adverse Event Trigger Tool’ modified for dental clinic use with that of a review of randomly selected electronic patient records. The study sites were dental practices where undergraduate dental students provide treatment under supervision.

The dental trigger tools were described as follows:

1. Development of infections
2. Failure of complex procedures (for example implant failures)
3. Multiple visits - more than six completed visits during the six month review period, or patients needing referrals to other specialists.

The electronic records were analysed for the above triggers via queries to the system. Each record was also analysed by two dentists in order to determine whether any adverse events had occurred by reading the narrative in the notes. The assessors also rated severity. For further analysis, 50 records were randomly selected and analysed per teaching dental practice to ascertain whether the findings from these notes were representative of those from the larger sample.

In total, 8,931 patients were seen during the six month study period and the computer system matched 315 cases with the triggers. Combining all triggers from the electronic notes search compared to the assessor’s findings from the notes, they found that the trigger tools had a positive predictive value of 0.5 (0.45-0.56, 95 % CI). The 50 randomly selected notes had a value of 0.34 (0.22-0.48, 95 % CI). In terms of severity, the assessing dentists graded the adverse events observed. The majority were found to have caused temporary harm, with one patient requiring hospitalisation due to infection and nine having permanent harm (failed implants that were not replaced).

This paper had clear aims and results; the processes were described in detail and the analysis was suitably rigorous. As the system for identifying the trigger tools and adverse events was computerised, the authors attempted to compare the efficacy of the system to their own analytic skills. We note that there was no assessment of the inter-rater reliability of the two assessing dentists. The study concluded that the dental clinic trigger tool was more effective in identifying adverse events than was a review of randomly selected records.

## Discussion

As far back as 2004, checklists were being introduced into dental hospitals in an attempt to reduce the incidence of wrong tooth extractions [66] with further work conducted focusing on the checklist systems used by the airline industry for ensuring passenger safety [67]. There are, of course, fundamental differences between the cockpit of an aircraft and a dental surgery or operating theatre; one is the fact that the dentist will not share the same fate as the patient in the event of a catastrophic error occurring. Many of these checklists share characteristics from the WHO surgical safety checklist devised in 2008 and used in UK hospitals from 2010 [63].

Two included studies on the use of checklists [57, 62] demonstrated a decrease in the number of erroneous extractions following the introduction of checklists. These findings are in contrast to the findings of the American Medical Association review of safety in ambulatory care which concluded that interventional studies had shown no difference or had negative impacts on safety [68]. However, these studies cannot definitively show that the introduction of the checklist was the sole explanation for the noted reductions in erroneous tooth extractions, as there are many variables in the workings of a department that could have influenced this.

A separate article by the same authors who published on the use of checklists [43] suggests seven steps for improving patient safety, by ensuring that risk management is applied to clinical dentistry. These are summarised in Table 3.

**Table 3:** Seven steps to improve patient safety in dentistry

Promotion of a Culture of Patient Safety in dental care.
Creating an organizational structure for the management of dental care risks.
Developing tools for the identification, analysis and assessment of risks related with dental care.
Establishing lines of information on adverse events.
Establishing measures to prevent health care risks by elimination or reduction.
On-going training of professionals on Patient Safety.
Research in the field of Dental Patient Safety.

These steps are similar to those suggested in the Annals of Internal Medicine in 2013 [69]; this paper describes the patient safety strategies which are ready for adoption based on best available evidence and also recommends priority areas of research to be pursued in order to answer outstanding questions on how to improve safety, including assessment of the impact of interventions designed to improve safety. They are also similar to the steps advised by the National Patient Safety Agency in the UK. These included the following: promote incident reporting, involve patients in the development of interventions and implement solutions known to prevent harm [70].

The use of reporting systems is positively encouraged in healthcare services [5]. One of the key benefits of using a reporting system is that it can be used as a learning tool to improve patient safety [12, 71], therefore allowing healthcare workers to learn from other people’s mistakes as well as their own. Significant event reviews are an important tool for learning from incidents when they do occur [72]. A recent British study described their use amongst General Medical Practitioners; they found that practitioners exercise selectivity over which errors/incidents to report. They also noted that practitioners were reluctant to report errors that could be addressed within their own practices. The competitive nature of primary care was also found to be relevant, with practitioners keen not to undermine patient confidence in their services [73]. This aspect of patient safety will be highly relevant to primary care dentists, as dental practices are disparate small businesses [74].

The use of electronic notes has been trialled previously in the medical literature [75] and recommended for use by dentists [46]. A recent review concluded that IT implementation in healthcare leads to improvements in quality, safety and efficiency [76].

This is the first systematic review of the literature on interventions used to improve patient safety in all areas of dentistry. The review demonstrates a noticeable lack of literature showing evidence of patient safety interventions leading to improvements. A number of tools have been identified but not verified from our literature searches: checklists, reporting systems, trigger tools, and the use of electronic notes. The authors are fully aware that this research paper is secondary research and that its main purpose is to encourage further research into patient safety in dentistry.

On a positive note, there are an increasing number of publications on patient safety in dentistry and it is an active area of research. The main issue with the studies included in this review are that they suggest interventions, but do not trial them. There was only one study that could be described as a randomised control trial [55] and this did not prove that the intervention had an impact on patient safety. The remainder were a mixture of case series, opinion pieces, educational pieces and observational studies.

Patient safety is a complex and multifactorial issue [11] with the potential for patient safety incidents to occur as a result of almost any interaction with the healthcare system [77]. In dentistry, the skill, experience and up-to-date knowledge of the practitioner have traditionally been relied upon in order to protect patients from harm when receiving treatment. Regulations, standards (from regulating bodies) and guidelines aim to maintain safe and effective care for dental patients [78]. Many harm prevention measures are already integrated into dental care in the fields of radiology, infection control, prescribing and teamwork. Several aspects of these measures are mandated by law in many countries [17]. The knowledge, attitudes and practices of dentistry vary between nations. Infection control practices are known to vary between countries, with certain countries lacking the resources to allow infection control measures to be robust; this also applies to the availability of vaccinations for dental clinical staff [79].

One widely available, but commonly underused measure for maintaining patient safety, is rubber dam. This simple intervention is known to control cross-infection and to protect patients from ingestion or aspiration of foreign bodies or irritants used in dentistry [80].

This review found there were no independently verified, well-validated tools in use that can lead to improvements in patient safety specific to dentistry; it also reveals other deficiencies in the evidence base:

- There is little understanding of basic epidemiology of patient safety in dentistry: we do not know if patient safety is a problem in dentistry, and if it is, the nature and size of the problem,
- A lack of an agreed conceptual understanding of patient safety in dentistry – how different is it from other areas of health care e.g. primary medical care and how it fits into the broader field of quality in dentistry,
- Little understanding of the views of patients on this issue.

Therefore, a collaborative approach is required. Dental researchers must work with researchers from other areas of primary care to develop concepts for improving patient safety using common methods and an agreed taxonomy. It is also important that priority areas for patient safety are identified by analysing available data on adverse events and the consequences of these, along with the production of clinical care guidelines addressing these issues.

## Conclusions

This systematic review finds that the only interventions in dentistry that reduce or minimise adverse events are surgical safety checklists.

It should be clear from reading this review that research into patient safety in dentistry is in its infancy, as it is in other aspects of ambulatory healthcare [68]. Healthcare quality is made up of multiple domains including safety, effectiveness, patient-centeredness, timeliness, efficiency and equitability [81]. It is important that tools developed to improve patient safety are adapted and customised for different healthcare settings so that they are appropriate to the patients and staff in those areas. We must be aware that not all tools and techniques found in other industries, such as aviation, are appropriate for transfer to healthcare settings [12].

All of the papers included in this review mention both the need for further research into patient safety in dentistry and the importance of educating practitioners in how to improve patient safety. This review echoes these calls for further research; we have demonstrated that a systematic approach to the investigation of patient safety in dentistry is required. The profession, in collaboration with patients, needs to develop a common understanding of the concept; we need to understand the epidemiology of patient safety in dentistry in different contexts. Furthermore, systematically developed tools need to be produced and appropriately trialled to minimise harm to patients.

## Additional files

Additional file 1: **PRISMA 2009 Checklist.** (PDF 106 kb)
Additional file 2: **Search Strategy (completed on 28.2.2014)** (DOCX 15 kb)
Additional file 3: **Characteristics of excluded studies.** (DOC 148 kb)

## Abbreviations

CASP: critical appraisal skills programme
CI: confidence interval
COPD: cronic obstructive pulmonary disorder
IT: information technology
MeSH: medical subject headings
UK: United Kingdom
US: United States
USA: United States of America
WHO: World Health Organisation
