# Supplementary material for: Systematic review of patient safety interventions in dentistry
Source: BMC Oral Health. 2015 Nov 28;15:152. doi: 10.1186/s12903-015-0136-1 (PMC4662809; doi:10.1186/s12903-015-0136-1)
Supplement: Additional file 2: — Search Strategy (completed on 28.2.2014) (DOCX 15 kb) [file 12903_2015_136_MOESM2_ESM.docx]

## Appendix 2

### Search Strategy (completed on 28.2.2014)

**MEDLINE via OVID, EMBASE via OVID, HMIC via OVID, CINAHL via EBSCO and Web of Science Search Strategies (MESH terms)**

1. exp Specialties, dental/

2. Surgery, Oral/

3. Infection control, dental/

4. exp Endodontics/

5. Diagnosis, oral/

6. Oral surgical procedures/

7. Operative dentistry/

8. Dental equipment/

9. Dental care/

10. Anesthesia, dental/

11. (dentist$ or (dental adj5 clinic$) or (dental adj5 office$)).ti,ab.

12. exp Dentists/

13. (dental adj practitioner$).ti,ab.

14. GDP.ti,ab.

15. exp Dental auxiliaries/

16. (dental and (hygienist$ or therapist$)).ti,ab.

17. (oral adj (surgeon$ or surger$)).ti,ab.

18. ("oral health practitioner" or "dental assistant$" or "dental auxil$" or "dental hygiene practitioner$" or "community dental health co-ordinator$" or "oral health co-ordinator$").ti,ab.

19. or/1-18

20. Patient safety/

21. Patient harm/

22. Medical error/

23. (administration adj error$).ti,ab.

24. (dispens$ adj error$).ti,ab.

25. (medica$ adj (mistake$ or error$)).ti,ab.

26. ((prescription$ or prescrib$) adj (error$ or fault$)).ti,ab.

27. (malpractice or safety or harm$).ti,ab.

28. (adverse adj (effect$ or event$ or reaction$)).ti,ab.

29. (never adj event$).ti,ab.

30. or/20-28

31. exp Healthcare evaluation mechanisms/

32. (scale$ or survey$ or questionnaire$ or instrument$ or indicator$ or outcome$ or "patient experience$" or "practice guideline$" or "quality assurance$" or tool$).ti,ab.

33. Practice guideline/

34. or/31-33

35. 19 and 30 and 34
