# Supplementary material for: Systematic review of patient safety interventions in dentistry
Source: BMC Oral Health. 2015 Nov 28;15:152. doi: 10.1186/s12903-015-0136-1 (PMC4662809; doi:10.1186/s12903-015-0136-1)
Supplement: Additional file 3: — Characteristics of excluded studies. (DOC 148 kb) [file 12903_2015_136_MOESM3_ESM.doc]

## Appendix 3

Characteristics of excluded studies:

| **Study** | **Reason for exclusion** |
| --- | --- |
| Vamnes 2004 | Describes patch testing for patients affected by adverse reactions to dental materials |
| Milgrom 1996 | Questionnaire on frustrating patient visits and their correlation with malpractice claims |
| Rickard 2004 | This is an opinion piece on the importance of training temporary dental nurses in order to maintain patient safety |
| Umesan 2012 | A set of guidelines based on a single practitioner’s experience on how to avoid orthodontic materials and equipment being ingested or aspirated during treatment. |
| Abdel-Galil 2010 | This is a letter to the editor of a journal highlighting the importance of introducing correct site surgery checklists into OMFS units |
| Chicago Dental Assistant 1994 | These two papers are fliers for courses aimed at improving health and safety for dental staff, published in the same edition of a journal for dental assistants. |
| Seager 2013 | Use of airline style checks in OMFS operating theatres |
| Spicer 2008 | Audit into use of computerised notes. No intervention is tested |
| Donnelly 2004 | Opinion piece on how to maintain health and safety in dental practices |
| Mellor 1995 | Questionnaire on practitioner frustration with patents. Similar to |
| Bluebond-Langner 2010 | Analysis of serious events in oral surgery and advice on how to break this news to patients and their families |
| Conrad 1998 | Article focuses on malpractice rather than patient safety |
| Donaldson 2012 | Discussion of the safety features of a nitrous oxide sedation machine |
| Brignardello-Petersen 2013 | Looks at the introduction of clinical guidelines in dentistry and suggests that they should be introduced with enhanced methodologies |
| Dym 2008 | Questionnaire for insurance firms on litigation |
| Decani 2013 | Advocates good medical history recording in patient notes. Abstract in English, rest of article in Italian |
| Amerena 2011 | Advice on the storage of sterile instruments in dental practices |
| Carpenter 1991 | Advice on implementing state guidelines on health and safety in the workplace |
| Iglesias-Linares 2013 | Use of a checklist to reduce flawed decision making by students of orthodontics. Article in Spanish; English abstract. |
| Gjerdet 2003 | This is a conference poster which features some of the data from the study by Lygre and colleagues |
| Kukadia 2011 | Conference abstract, unable to gather any further information from the authors |
| Stern 2011 | Conference abstract, unable to gather any further information from the authors |
| Perea-Perez 2011b | This is an educational piece which does not mention an intervention for improving patient safety |
| Valenza 1994 | This single author paper advises practitioners on how to organise their case notes in a consistent manor to reduce the risk of litigation and to conform the regulations of insurers. There is no intervention trialled here. |
| Hiivala 2013 | This paper describes a questionnaire that was sent to Finnish dentists asking for their experiences of patient safety incident prevention, reporting systems and feedback from safety incidents in their practices. There is no patient safety intervention trialled here. |
| Diaz-Flores-Garcia 2014 | This paper describes a checklist for use when performing endodontic treatments under local anaesthesia. There is no trialling of the checklist however, and no data is presented demonstrating any improvements in patient safety outcomes following its introduction. |
| Knepil 2008 | Analyses the results of a questionnaire that was sent to both Oral & Maxillofacial Surgeons and Anaesthetists to question their use of throat packs for oral surgical procedures performed under general anaesthesia. The authors designed an ‘organisational accident model’ relating to the retention of throat packs, however, there is no tool trialled in the paper. |
| Knepil 2013 | This study describes an audit in which the authors asked surgeons and patients about their thoughts on marking of the skin prior to oral surgical procedures under general anaesthesia. The cheek was preferred for marking, although the forehead was preferred in patients with significant amounts of facial hair. The authors state that they have had not had any erroneous tooth extractions occur in their unit since the introduction of site marking, however they do not have any figures or timeframes for comparison and analysis in our systematic review. |
| Raja 2014 | The authors describe an educational module introduced to the undergraduate curriculum in the dental school at Chicago, Illinois. The module includes teaching students about adverse events and how to communicate with patients about these when they occur. The student’s knowledge about adverse events was improved as demonstrated by pre and post-test scores. No intervention is trialled and there is no reduction in adverse events demonstrated, this is an educational piece. |

1. Vamnes JS, Lygre GB, Gronningsaeter AG, Gjerdet NR: **Four years of clinical experience with an adverse reaction unit for dental biomaterials**. *Community Dentistry and Oral Epidemiology* 2004, **32**(2):150-157.

2. Milgrom P, Cullen T, Whitney C, Fiset L, Conrad D, Getz T: **Frustrating patient visits**. *Journal of Public Health Dentistry* 1996, **56**(1):6-11.

3. Rickard GD: **An outline of appropriate risk management in the use of temporary dental nursing staff in practice**. *British Dental Journal* 2004, **197**(11):674-679.

4. Umesan UK, Chua KL, Balakrishnan P: **Prevention and management of accidental foreign body ingestion and aspiration in orthodontic practice**. *Therapeutics and Clinical Risk Management* 2012, **8**:245-252.

5. Abdel-Galil K: **The WHO surgical safety checklist: are we measuring up?** *British Journal of Oral & Maxillofacial Surgery* 2010, **48**(5):397-398.

6. **Setting up and maintaining a dental office safety program**. *Dental assistant (Chicago, Ill : 1994)* 2005, **74**(6):40-42.

7. **Putting it all together. An effective dental office safety program**. *Dental assistant (Chicago, Ill : 1994)* 2005, **74**(6):42-44.

8. Seager L, Smith DW, Patel A, Brunt H, Brennan PA: **Applying aviation factors to oral and maxillofacial surgery--the human element**. *British Journal of Oral & Maxillofacial Surgery* 2013, **51**(1):8-13.

9. Spicer R: **'Bytes and bites'--using computerized clinical records to improve patient safety in general dental practice**. *Dental Update* 2008, **35**(9):614-616, 618-619.

10. Donnelly T: **Dental practice safety--an inspector's viewpoint**. *Journal of the Irish Dental Association* 2004, **50**(2):77-78.

11. Mellor AC, Milgrom P: **Dentists Attitudes toward Frustrating Patient Visits - Relationship to Satisfaction and Malpractice Complaints**. *Community Dentistry and Oral Epidemiology* 1995, **23**(1):15-19.

12. Bluebond-Langner R, Rodriguez ED, Wu AW: **Discussing adverse outcomes with patients and families**. *Oral & Maxillofacial Surgery Clinics of North America* 2010, **22**(4):471-479.

13. Conrad DA, Milgrom P, Whitney C, O'Hara D, Fiset L: **The incentive effects of malpractice liability rules on dental practice behavior**. *Medical Care* 1998, **36**(5):706-719.

14. Donaldson M, Donaldson D, Quarnstrom FC: **Nitrous oxide-oxygen administration: when safety features no longer are safe**. *Journal of the American Dental Association* 2012, **143**(2):134-143.

15. Brignardello-Petersen R, Carrasco-Labra A, AbdelAziz A, Hartshorne J, Azarpazhooh A: **Rigor of development of clinical practice guidelines in dentistry**. *BMJ Quality and Safety* 2013, **22**:A63.

16. Dym H: **Risk management techniques for the general dentist and specialist**. *Dental Clinics of North America* 2008, **52**(3):563-577, ix.

17. Decani S, Baruzzi E, Martini V, Ficarra G, Lodi G: **Dentistry adverse drug reactions and drug interactions. [Italian] Reazioni awerse e interazioni farmacologiche di interesse odontoiatrico**. *Dental Cadmos* 2013, **81**(3):125-135.

18. Amerena VC: **Safe storage times for sterile instrument packs**. *Australasian Dental Practice* 2011:100-102.

19. Carpenter KL: **Safety standards for the dental office**. *The Journal of the Michigan Dental Association* 1991, **73**(9):36-38.

20. Iglesias-Linares A, Yáñez-Vico RM, Solano-Reina E: **Uso de una checklist para reducir los errores cognitivos en la enseñanza práctica odontológica**. *FEM: Revista de la Fundación Educación Médica* 2013, **16**(1):31-36.

21. Gjerdet NR, Bjorkman L: **A specialised national reporting system of adverse reactions to dental materials**. *Journal of Dental Research* 2003, **82**:530-530.

22. Lygre GB, Gjerdet NR, Gronningsaeter AG, Bjorkman L: **Reporting on adverse reactions to dental materials - intraoral observations at a clinical follow-up**. *Community Dentistry and Oral Epidemiology* 2003, **31**(3):200-206.

23. Kukadia R, Dajani H, Sedani P, Hardee P, Bridle C, Ali N, Nasser N: **Implementation of the "wHO Surgical Safety Checklist" in an outpatients setting - Minimising the risk for error**. *International Journal of Oral and Maxillofacial Surgery* 2011, **40 (10)**:1138.

24. Stern S, Dajani H, Bridle C: **An audit of a Surgical Safety Checklist for oral biopsies**. *Oral Oncology* 2011, **47**:S155.

25. Perea-Perez B, Santiago-Saez A, Garcia-Marin F, Labajo-Gonzalez E, Villa-Vigil A: **Patient safety in dentistry: Dental care risk management plan**. *Medicina Oral Patologia Oral Y Cirugia Bucal* 2011, **16**(6):E805-E809.

26. Valenza JA: **Medical risk report: improving patient management and record keeping through a problem-oriented approach**. *Journal of the Greater Houston Dental Society* 1994, **65**(9):46-48; quiz 49.

27. Hiivala N, Mussalo-Rauhamaa H, Murtomaa H: **Patient safety incident prevention and management among Finnish dentists**. *Acta Odontologica Scandinavica* 2013, **71**(6):1663-1670.

28. Diaz-Flores-Garcia V, Perea-Perez B, Labajo-Gonzalez E, Santiago-Saez A, Cisneros-Cabello R: **Proposal of a "Checklist" for endodontic treatment**. *Journal of clinical and experimental dentistry* 2014, **6**(2):e104-109.

29. Knepil GJ, Blackburn CW: **Retained throat packs: results of a national survey and the application of an organisational accident model**. *The British journal of oral & maxillofacial surgery* 2008, **46**(6):473-476.

30. Knepil GJ, Harvey CT, Beech AN: **Marking the skin for oral surgical procedures: improving the WHO checklist**. *The British journal of oral & maxillofacial surgery* 2013, **51**(5):413-415.

31. Raja S, Rajagopalan CF, Patel J, Van Kanegan K: **Teaching dental students about patient communication following an adverse event: a pilot educational module**. *Journal of dental education* 2014, **78**(5):757-762.
